# Residual disease as a therapy-remodeled ecosystem: spatial niches, tumor persistence, and relapse in solid tumors

**DOI:** 10.3389/fonc.2026.2004743

**Published:** 2026-10-27

**Authors:** Nataša Prvulović Bunović, Olivera Ivanov, Saima Korać, Nikola Denda, Ivana Majkić, Nemanja Maletin

**Affiliations:** 1 Faculty of Medicine, University of Novi Sad, Novi Sad, Serbia; 2 Oncology Institute of Vojvodina, Sremska Kamenica, Serbia; 3 University Clinical Center of Vojvodina, Novi Sad, Serbia

**Keywords:** cancer relapse, drug-tolerant persister cells, microenvironmental niche, molecular residual disease, spatial transcriptomics, therapy resistance, tumor dormancy, tumor microenvironment

## Abstract

Residual disease comprises the malignant burden that remains after treatment, including low-volume lesions and clinically occult reservoirs. Surviving cells encounter a tissue environment altered by cell death, inflammation, repair, stromal activation, vascular injury, and metabolic stress. These changes can support spatially restricted persistence, but co-localization alone does not establish a protective niche. This narrative review links therapy-associated tissue remodeling to the location and state of residual cells and to the timing of potential relapse-directed intervention. We distinguish molecular residual disease, treatment-tolerant persister states, dormancy, and stable resistance, and separate selection of pre-existing populations from experimentally demonstrated state induction. Across chemotherapy, radiotherapy, targeted therapy, endocrine therapy, and immunotherapy, we distinguish human observational or trial evidence from mechanistic findings in mouse models, organoids, and cell culture. We organize niche evidence into spatial enrichment, association with a residual-cell state, selective perturbation, and an effect on reactivation or relapse. Examples illustrate dose-dependent radiation responses, multifactorial stromal protection, context-dependent immune surveillance, and collateral metabolic vulnerabilities. Longitudinal spatial profiling, lineage analysis, spatial pharmacology, and clinically informative circulating tumor DNA studies offer complementary information; molecular detection does not identify a particular tissue niche or directly demonstrate viable residual cells. Four therapeutic windows are proposed as research strategies rather than a validated clinical classification. Their utility depends on the interaction being required, the drug reaching the relevant region, and acceptable effects on normal repair and tumor-restraining functions. Limited paired human sampling and the compressed timescale of experimental models remain major constraints on translation.

## 1 Introduction

Many solid tumors undergo substantial radiographic or pathological regression without complete eradication. Surviving cells may occupy measurable lesions, microscopic tumor islands, or dispersed reservoirs. When conventional assessment shows no disease, circulating tumor DNA (ctDNA) can provide a molecular signal associated with recurrence risk; it does not directly localize or demonstrate viable residual cells. Relapse requires malignant cells to survive and subsequently generate net growth. Classical resistance models explain survival through pre-existing or acquired alterations affecting drug targets, bypass signaling, apoptosis, DNA repair, or immune recognition. These mechanisms do not fully explain why genetically similar cells in adjacent regions can experience different treatment outcomes or why transient tolerance may reverse after drug withdrawal.

A solid tumor is a spatially organized tissue in which malignant cells interact with fibroblasts, extracellular matrix (ECM), endothelial and perivascular cells, macrophages, neutrophils, dendritic cells, lymphocytes, nerves, and organ-specific parenchyma. Oxygen, nutrients, metabolites, cytokines, and therapeutic agents are distributed unevenly through this tissue (1, 2). Treatment changes both the malignant population and this local ecology. Cytotoxic injury activates repair programs; targeted therapy changes the signals exchanged between oncogene-dependent cells and stroma; radiotherapy alters vessels and inflammatory signaling; and immunotherapy applies selection at the level of antigen presentation, trafficking, and effector function.

The residual phase can represent a population bottleneck and an opportunity in selected settings. Lower burden does not guarantee targetability: survivors may retain diverse states, include stable resistant clones, or depend on functions shared with normal tissue. Some cells are quiescent, whereas others cycle slowly or intermittently. Local signals may acquire greater importance during treatment, and prolonged survival can permit further adaptation. Whether a dependency is enriched specifically during persistence must be tested against pretreatment and recurrent disease rather than assumed.

Spatial studies show nonrandom residual-cell organization in experimental tumors. Peristromal regions in ALK-positive lung cancer models and residual islands in *Brca1/Trp53*-deficient mouse mammary tumors connect treatment, location, cell state, and regrowth (3, 4). **Figure 1** presents a proposed temporal model, and **Table 1** compares candidate niche classes that may coexist within the same region.

**Figure 1:**
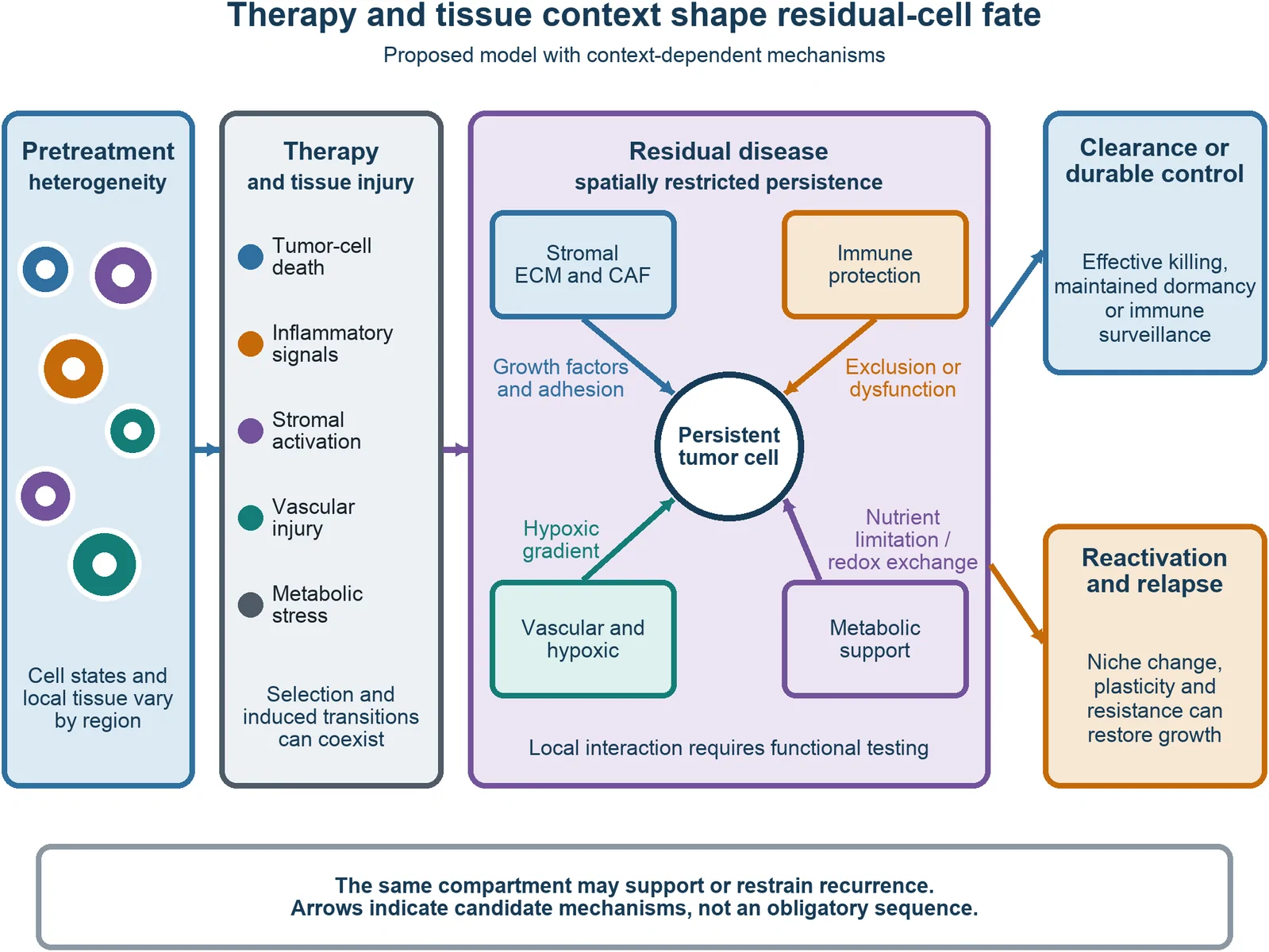
Proposed model of therapy-associated remodeling and residual-cell fate. Treatment can select pre-existing populations, induce adaptations, and alter tissue composition. Labeled arrows indicate candidate local drivers: growth-factor and adhesion signaling, exclusion or effector dysfunction, hypoxic gradients, and nutrient limitation or redox exchange. The stromal, immune, vascular, and metabolic configurations can overlap. Clearance, controlled persistence, and regrowth are possible outcomes rather than obligatory consecutive stages. Arrow labels describe mechanistic classes and do not assert causal validation in every tumor type; the evidence levels in **Table 1** apply to individual studies.

**Table 1:** Candidate functional classes of residual-disease niches and evidence required to support their interpretation.

Niche class	Dominant components	Candidate function	Evidence questions	Intervention principle	Main risk
Peristromal	CAFs, matrix, growth factors, integrins	Bypass signaling or adhesion survival	L1⁠–⁠L2: proximity and state; L3: perturb signaling; L4: test regrowth	Target a demonstrated interaction or shared vulnerability	Redundancy; loss of tumor restraint
Immune protected	Myeloid cells; excluded or dysfunctional T cells	Impaired recognition, access, or killing	L1⁠–⁠L2: contacts and immune state; L3: restore killing; L4: test clearance/relapse	Restore the measured immune bottleneck	Loss of repair or effective antigen presentation
Vascular and hypoxic	Vessels, perivascular cells, hypoxic tumor cells	Uneven exposure or stress adaptation	L1⁠–⁠L2: exposure/hypoxia maps; L3: alter delivery or signaling; L4: test regrowth	Correct a measured exposure deficit or dependence	Worsened hypoxia; normal-vessel injury
Metabolic	Nutrient/redox exchange; adapted cells	Fuel support or antioxidant buffering	L1⁠–⁠L2: metabolite–state map; L3: isotope/rescue tests; L4: test outgrowth	Exploit a verified metabolic interaction	Immune and normal-cell toxicity
Invasive or organ specific	Budding/EMT-like cells; organ parenchyma	Site-dependent survival or plasticity	L1⁠–⁠L2: paired state/location; L3: organ-informed perturbation; L4: test relapse	Match target to state and metastatic site	Association mistaken for a causal niche

Evidence levels are L1, spatial enrichment; L2, association with a residual-cell state; L3, selective perturbation with an effect on persistence; and L4, an effect on reactivation or relapse. These are cumulative questions rather than established grades for entire niche classes. The evidence column describes what should be tested, not what all cited studies have demonstrated. Human clinical efficacy requires additional validation. CAF, cancer-associated fibroblast; ECM, extracellular matrix; EMT, epithelial–mesenchymal transition; Treg, regulatory T cell.

A recent narrative review has synthesized immune exclusion and exhaustion in relation to microenvironmental therapy resistance (5). The specific contribution of this review is to connect three questions usually considered separately: how treatment remodels tissue, where and in which states malignant cells persist, and when an intervention against the supporting interaction might alter relapse. We organize evidence by treatment interval, spatial context, causal strength, and experimental system. A residual niche is therefore a candidate local configuration until functional evidence shows that it promotes survival, regrowth competence, or escape from clearance. This framework complements descriptions of persister-cell programs and dormancy by specifying the evidence needed to connect those states to a therapeutically relevant tissue interaction.

## 2 Review approach

This targeted narrative review used PubMed, publisher full-text records, and reference lists of relevant articles. The initial searches were conducted from 31 August to 4 September 2026; focused searches and source checks for this revision were performed through 12 October 2026. Search concepts combined residual disease, drug-tolerant persisters, dormancy, or relapse with microenvironment, fibroblast, extracellular matrix, immune exclusion, myeloid cell, vascular, hypoxia, or metabolism, and with chemotherapy, radiotherapy, targeted therapy, endocrine therapy, or immunotherapy. Additional searches covered spatial and single-cell profiling, lineage tracing, pharmacology, and ctDNA. No fixed starting publication year was imposed because landmark experiments were needed to interpret recent findings.

Studies were selected for a defined treatment context, identifiable sampling interval, and a mechanistic, spatial, or clinically relevant outcome. Primary peer-reviewed investigations support mechanistic and clinical claims; reviews are retained only for general conceptual context. Randomized human trials support clinical utility in their tested settings, observational human tissue supports localization or association, and selective model perturbations support causality within that model. These categories were not treated as interchangeable or ranked by sample size alone. Selection was iterative and purposive, without duplicate screening, a registered protocol, formal risk-of-bias scoring, or pooled analysis.

## 3 A working vocabulary for residual disease

### 3.1 Residual disease and molecular residual disease

Residual disease denotes the malignant burden remaining after treatment, whether clinically measurable or below conventional detection. Minimal residual disease describes low volume and is not used here as a mechanistic or molecular definition. Residual viable tumor in a resection specimen differs from occult cells remaining in the patient after apparently complete surgery. A small metastatic lesion persisting during treatment also differs from a positive postoperative ctDNA assay. We use molecular residual disease specifically for tumor-derived molecular material detected when conventional assessment shows no disease; this is an assay-defined state rather than direct evidence of viability, location, or niche dependence.

Postoperative ctDNA identifies recurrence risk and has prospectively guided adjuvant treatment in selected settings, including stage II colon cancer and muscle-invasive bladder cancer (6, 7). Its utility remains context-dependent: stage III colon cancer ctDNA-guided de-escalation did not meet the prespecified noninferiority boundary (8). These clinical trials address treatment selection based on molecular status, not identification or elimination of a spatial niche. ctDNA cannot determine whether its source cells are dormant or proliferating, which tissue compartment contains them, or whether a candidate microenvironmental target is present.

### 3.2 Drug tolerance, persistence, dormancy, and resistance

Drug tolerance describes survival during exposure without requiring stable resistance. Here, treatment-tolerant persister cells denote a surviving state or subpopulation under drug pressure whose reduced sensitivity is potentially reversible; the term is not a synonym for every residual cell or for a clone with established genetic resistance. A reversible chromatin-mediated state was demonstrated in cancer cell cultures (9). Lineage-resolved culture experiments subsequently showed that pretreatment programs and treatment-associated transitions can coexist, with cycling and noncycling persisters using distinct programs (10).

Dormancy can refer to single-cell quiescence or population equilibrium between proliferation and loss. Not all residual disease is dormant. In colorectal cancer patient-derived experimental models, chemotherapy-associated cells entered a reversible diapause-like program (11). Melanoma patient-derived xenografts contained multiple neural crest-like, invasive, and starved residual states (12). Human lung cancer single-cell profiles identified an alveolar-regenerative program during treatment response, but sampling different clinical states did not establish the ancestry of every residual population (13). Resistance denotes reduced treatment effect that permits survival or growth and may be genetic, epigenetic, or environmentally mediated. Tolerance can precede stable resistance, but the sequence is not obligatory.

### 3.3 Selection, induction, and ecological rescue

An on-treatment cell state may arise through selection of a rare pretreatment population, induction of a new state, altered trafficking, or differential loss of neighboring cells. These processes are not mutually exclusive. High-complexity barcoding, repeated sampling, and lineage-resolved single-cell analysis can distinguish clonal selection from reversible state transitions (10, 14). A state enriched after treatment should not automatically be labeled therapy-induced when it was present below the detection threshold beforehand.

Ecological rescue describes survival conferred by a local environment despite high cell-intrinsic drug sensitivity. It is supported when spatially restricted exposure, contact, or paracrine signaling changes survival and selective perturbation reverses that effect. Because several mechanisms may operate in one neighborhood, failure of a single-pathway inhibitor does not exclude ecological rescue.

### 3.4 Evidence levels for a residual niche

We distinguish four levels of biological evidence: L1, reproducible spatial enrichment of surviving cells near a compartment; L2, association of that neighborhood with a defined residual-cell state or function; L3, selective perturbation of the interaction that changes persistence, ideally with rescue or an orthogonal test; and L4, an effect on reactivation or relapse. A level-4 result in a mouse establishes causality in that system, not human clinical efficacy. A treatment that reduces bulk tumor growth without testing the local interaction is also insufficient. **Table 1** uses these levels to state what must be tested for each candidate class.

## 4 Therapy as an architect of residual niches

### 4.1 Chemotherapy: tumor killing and wound repair occur together

Chemotherapy reduces malignant burden while injuring nonmalignant cells. Paired human prostate specimens and stromal-cell experiments linked genotoxic damage to NF-κB-dependent WNT16B secretion and resistance-promoting effects (15). Colorectal cancer fibroblast and xenograft experiments implicated treatment-associated IL-17A signaling (16). Platinum-exposed mesenchymal stromal cells released fatty acids that protected tumors in mice (17), and clearance of therapy-induced senescent normal cells reduced recurrence in chemotherapy-treated mice (18). These interventions support particular repair mechanisms; an increase in fibroblast abundance or a repair signature alone would not establish new-state induction or protection.

Drug-stressed melanoma and lung adenocarcinoma cells also produced secreted signals that supported neighboring sensitive and resistant populations in experimental cultures and mouse tumors (19). The experiments establish a treatment-dependent secretory response, not that every post-treatment secretome is newly induced or supports regrowth in patients.

Longitudinal profiling of syngeneic *Brca1/Trp53*-deficient mouse mammary tumors showed enrichment of an EMT-associated population already detectable before treatment, alongside depletion of proliferative and hypoxic compartments. The mice received a single intravenous cisplatin dose of 6 mg/kg or TAC comprising doxorubicin 2.5 mg/kg intravenously, docetaxel 12.5 mg/kg intraperitoneally, and cyclophosphamide 120 mg/kg intraperitoneally; residual tumors were sampled at 7 or 12 days. Recurrent tumors recovered much of the primary composition, but neither the cells seeding recurrence nor *de novo* EMT induction were lineage-resolved. Human residual breast cancer specimens corroborated selected spatial signatures rather than the full mouse trajectory (4).

### 4.2 Radiotherapy: immunogenicity, vascular injury, and repair

In mouse tumor models, radiotherapy activated cytosolic DNA sensing and type I interferon-dependent antitumor immunity (20). Vanpouille-Box and colleagues found that radiation-induced TREX1 expression sufficient to clear cytosolic DNA occurred at approximately 12–18 Gy per fraction, depending on the mouse or human carcinoma cell model. Three consecutive daily fractions of 8 Gy supported interferon signaling and checkpoint-blockade-associated abscopal responses in the TSA mouse model, whereas a single 20-Gy exposure induced TREX1 and limited this response (21). These are experimental, model-dependent thresholds, not a universal human planning constraint or proof that 8 Gy × 3 is optimal for all tumors. Fraction size, sequence, target volume, and normal-tissue exposure matter alongside total dose.

In orthotopic human glioblastoma xenografts in immunodeficient mice, a single 15-Gy exposure reduced perfusion and increased hypoxia, followed by HIF-1-associated recruitment of bone-marrow-derived CD11b-positive myelomonocytic cells, including monocytes/macrophages. These cells supported vascular recovery rather than being identified generically as endothelial precursors. Blocking the CXCL12–CXCR4 recruitment axis prevented regrowth after 15 Gy or five daily 2-Gy fractions in the tested models. Increased CD11b-positive infiltration in paired human recurrent glioblastomas was observational corroboration, not clinical proof of the intervention (22).

### 4.3 Targeted and endocrine therapy: pathway suppression creates new dependencies

Fibroblast-derived HGF reduced RAF-inhibitor sensitivity in melanoma co-cultures, with supporting patient observations (23). BRAF inhibition remodeled fibroblast–matrix signaling in experimental melanoma, strengthening integrin β1–FAK-dependent protection (24). Combined EGFR and MEK inhibition produced a YAP/TEAD-dependent senescence-like dormant state in EGFR-mutant lung cancer experimental models (25). These controlled exposure experiments support treatment-associated adaptation in their systems. The regenerative and inflammatory programs seen in human lung cancer profiles remain associations unless lineage or paired baseline evidence distinguishes induction from selection (13).

Endocrine therapy can leave clinically occult disease for prolonged intervals. In hormone-resistant breast cancer experimental models, extracellular-vesicle transfer of mitochondrial DNA restored oxidative phosphorylation and enabled escape from dormancy (26). MSK1 manipulation altered luminal differentiation and bone metastatic outgrowth in experimental models, while tumor MSK1 expression was associated with patient relapse (27). These results support specific mechanisms of outgrowth; they do not determine how frequently endocrine treatment induces a new state rather than selecting or sustaining a pre-existing one, nor explain all late recurrences.

In ALK-positive lung cancer co-cultures and xenografts, multiple growth-factor and adhesion signals supported peristromal survival during alectinib exposure. Regimens included 25 mg/kg alectinib by daily oral gavage, reduced to 20 mg/kg in selected combination experiments. Adding trastuzumab deruxtecan at 10 mg/kg every two weeks suppressed relapse in the tested xenografts, exploiting treatment-associated HER2 expression and bystander activity (3). Clinical histology supported a proliferation–stroma association, but the combination result was preclinical. These doses specify experimental exposure, not clinical equivalents; regional concentration and target engagement must be measured before extrapolation.

### 4.4 Immunotherapy: spatial exclusion and adaptive escape

Checkpoint-blockade failure can reflect defects in priming, trafficking, recognition, effector function, or memory. Chronic interferon exposure induced a multigenic resistance program in mouse experiments (28). Human melanoma profiles associated a malignant-cell program with T-cell exclusion, with functional support from experimental models (29). In a human urothelial cancer cohort, fibroblast TGF-β signaling was associated with stromal T-cell exclusion; combined TGF-β and PD-L1 blockade improved responses in mouse models (30). These designs distinguish some inducible mechanisms from baseline associations but do not show that all exclusion or dysfunction was created by immunotherapy. A persistent immune pattern should be called treatment-associated unless its origin is directly resolved.

## 5 Functional anatomy of protective niches

### 5.1 Fibroblast and extracellular-matrix niches

Cancer-associated fibroblasts (CAFs) regulate growth factors, cytokines, matrix composition, interstitial pressure, immune trafficking, and drug penetration. Their effects are state- and tissue-dependent. In pancreatic cancer models, depletion of α-SMA-positive myofibroblasts reduced survival and increased immunosuppression (31). Independently, genetic or pharmacological reduction of Sonic Hedgehog signaling diminished stroma but produced aggressive, undifferentiated, hypervascular tumors (32). These results show that a strategy based only on fibrosis abundance can remove tumor-restraining functions or release malignant cells from spatial constraint.

In human head and neck and pancreatic tumors, spatial pharmacobiology associated FAP-positive fibroblast and periostin-rich matrix neighborhoods with reduced delivery of panitumumab–IRDye800 (33). Head and neck participants received 50 mg 1–5 days before surgery. Pancreatic cohorts received 25–75 mg with 100 mg unlabeled panitumumab, or 50 mg labeled antibody alone, 2–5 days before surgery; drug–matrix analyses were restricted to a uniformly dosed 50-mg pancreatic subset. The low-dose imaging study measured regional exposure, not persistence under therapeutic antibody dosing. The association therefore identifies a delivery-related neighborhood, not a proven residual-cell dependency.

Matrix-rich neighborhoods should therefore be described by composition, architecture, signaling activity, and transport properties rather than by fibrosis alone. This distinction separates a physical delivery barrier from an adhesion-dependent survival signal and from a fibroblast-derived paracrine effect.

### 5.2 Myeloid niches and incomplete immune clearance

Macrophages, monocytes, neutrophils, and dendritic cells respond rapidly to treatment-induced injury. Their phagocytic, antigen-presenting, inflammatory, matrix-remodeling, angiogenic, and suppressive activities can coexist within one lesion. Post-treatment states therefore require functional and spatial definition beyond a binary M1/M2 label. The clinically relevant question is whether a specific on-treatment myeloid state promotes clearance of residual cells or supports their survival and reactivation.

In mouse breast tumor models, CCL5-mediated macrophage recruitment promoted recurrence and provided functional evidence beyond co-localization (34). In the syngeneic *Brca1/Trp53*-deficient mammary study, residual disease contained M2-annotated, SPP1-positive, and complement-high macrophage populations (4). That enrichment supplies state-associated spatial evidence, not proof that each macrophage population is required. Lineage-specific perturbation is needed to distinguish protection from debris clearance.

Suppressive macrophages can coexist with dendritic cells needed for IL-12 production and CD8-positive T-cell responses to chemotherapy (35). A residual-disease trial should therefore specify the targeted lineage and state, measure modulation in tumor tissue, and test whether antigen presentation and tumor-reactive T-cell function are preserved.

### 5.3 Lymphoid niches: exclusion, dysfunction, and surveillance

Adaptive immunity maintained occult transformed cells in equilibrium in mice (36). This establishes an experimental basis for immune restraint, not proof that every human residual reservoir is under such control. Surveillance requires T-cell access, antigen recognition, and retained killing function. Exclusion and dysfunction should be assessed separately because a lymphocyte-rich biopsy can still lack productive contact with malignant cells.

Primary human urothelial tumor analyses demonstrated stromal exclusion associated with fibroblast TGF-β signaling (30). In non-small-cell lung cancer specimens, a PD-1-high CD8-positive population retained tumor recognition but showed impaired classical effector cytokine production; its abundance was associated with benefit from PD-1 blockade in a small cohort (37). Dysfunction therefore does not imply complete absence of tumor reactivity or inevitable checkpoint resistance. Spatial contact, functional assays, and treatment outcome provide different evidence and should not be inferred from T-cell density alone.

### 5.4 Vascular, hypoxic, and metabolic niches

Vessels determine oxygen, nutrient, and drug delivery, but their relationship with persistence is not monotonic. Poorly perfused regions can receive less therapy and favor stress-adapted cells, whereas highly perfused sites may provide growth factors and access to activated stroma.

Culture and patient-derived experimental models identified lineage- and treatment-dependent metabolic programs in tolerant residual states (10, 12). Drug-tolerant or mesenchymal states were vulnerable to GPX4 inhibition in preclinical studies (38, 39). Hypoxia, nutrient availability, and antioxidant support are plausible modifiers, but a starved transcriptional signature alone does not prove that nutrient depletion created a niche. Metabolite measurement and selective rescue or inhibition are needed to establish the relevant interaction. Shared stress-response functions in immune and normal cells constrain translation.

Vascular niches can also restrain disease. Stable microvasculature maintained disseminated breast cancer cells in a quiescent state through endothelial thrombospondin-1, whereas sprouting endothelial tips supplied growth-promoting signals and supported outgrowth in experimental models (40). The vascular state, rather than vessel abundance alone, can therefore determine whether a perivascular site maintains dormancy or permits reactivation.

### 5.5 Invasive fronts, metastatic organs, and systemic niches

Organ-specific matrix and parenchyma can shape metastatic persistence. A single-center human cohort linked high tumor budding to worse 2-year overall survival in synchronous colorectal liver metastases, without an association with time to progression or 5-year survival (41). Because matched pretreatment tissue was unavailable, this is an example of the limits of single-timepoint pathology, not direct evidence of a therapy-remodeled residual niche.

Bone, liver, lung, brain, and peritoneal reservoirs encounter different endothelial, immune, and parenchymal programs. Evidence from one site cannot establish the same dependence at another.

Primary studies combined human response associations with transfer experiments in mice to link gut microbial composition to PD-1 blockade efficacy in epithelial cancers and melanoma (42, 43). These findings should not be generalized into a demonstrated acquired-resistance mechanism in MSI-high/mismatch-repair-deficient colorectal cancer. The gut microbiome is a systemic modifier of immune surveillance, not a local tissue niche, and these studies did not identify a specific residual reservoir.

## 6 From persistence to reactivation and relapse

Residual disease changes over time. Immediately after treatment, the dominant signals may be acute cell death, interferon, phagocytosis, and tissue repair. During continued suppression, surviving cells may enter quiescent, diapause-like, mesenchymal, regenerative, or metabolically adapted states. Reactivation can follow a change in the niche rather than a new tumor-cell mutation: in mouse models, sustained lung inflammation generated neutrophil extracellular traps that remodeled laminin and awakened dormant disseminated cells through integrin signaling (44).

Experimental regeneration from compositionally restricted residual disease illustrates why recurrence tissue may no longer contain the interaction relevant during response. A relapse biopsy cannot retrospectively establish that interaction without earlier sampling or lineage evidence.

Reactivation also changes the relative importance of extrinsic and intrinsic mechanisms. Early survival may be highly dependent on local stroma, whereas later clones with stable pathway reactivation can grow farther from the niche. Conversely, genetically resistant cells may remain dependent on matrix adhesion, antioxidant support, or immune exclusion. A linear model in which environmental tolerance simply gives way to genetic resistance is therefore too rigid. Relapse integrates both and may preserve vulnerabilities created during persistence.

## 7 Measuring residual niches

### 7.1 Longitudinal design and spatial sampling

A recommended informative design obtains pretreatment tissue, an early pharmacodynamic specimen, tissue near maximal response or surgery, and recurrence tissue where safe and feasible. This is an idealized set of complementary measurements, not a mandatory minimum for every trial. Same-lesion sampling reduces anatomical confounding, whereas multiregion sampling estimates heterogeneity. Studies should record exposure, dose intensity, interval since treatment, concomitant corticosteroids or antibiotics, and intervening procedures. **Table 2** sets out the rationale; **Table 3** distinguishes representative experimental systems and measured endpoints.

**Table 2:** Recommended longitudinal study design for causal analysis and clinical translation of a residual-disease niche.

Study element	Study question	Preferred measurement	Common interpretive error
Baseline	Was the residual state already present?	Multiregion tissue with lineage and spatial profiling	Calling enrichment after therapy *de novo* induction
Early on-treatment	Did the drug reach and engage the proposed region?	Spatial drug distribution, target engagement, proximal pharmacodynamics	Calling underexposure biological resistance
Maximal response	Which cells and neighborhoods persist?	Pathology, single-cell and spatial profiling, functional viability	Inferring residual biology from pretreatment or relapse tissue
Causal model	Is the interaction necessary for persistence or reactivation?	Selective perturbation in a model retaining the implicated compartment	Using a model that omits the causal compartment
Longitudinal biomarker	Can the niche be tracked during therapy?	Tissue-linked ctDNA, imaging, soluble or cellular marker	Treating a prognostic burden marker as a niche-specific biomarker
Clinical validation	Does intervention improve durable outcome?	Controlled trial with biological modulation and relapse endpoint	Relying on single-arm response or correlative association

Tissue timepoints should be selected according to safety, feasibility, and the biological question; repeated biopsy is not mandatory for every patient. Feasibility analyses should include nonevaluable samples and patients without tissue.

**Table 3:** Representative primary evidence across experimental systems.

Primary study	System and treatment context	Supported inference and main limit
Túrós et al. (4)	Syngeneic mouse mammary tumors; one cisplatin or TAC dose; residual sampling at 7/12 days; human tissue comparison	Spatial residual-state enrichment (L1⁠–⁠L2). No lineage-resolved recurrence or equivalent long human latency.
Desai et al. (3)	Human lung cancer co-cultures and mouse xenografts; daily alectinib; combination perturbations; supportive clinical histology	Functional protection tests; collateral targeting suppressed relapse. No single necessary stromal mediator isolated; patient efficacy unproven.
Vanpouille-Box et al. (21)	Mouse tumors and mouse/human carcinoma cells; 8 Gy × 3 versus 20 Gy × 1; TREX1 perturbation	Dose-dependent DNA sensing and immunogenicity. Model-specific threshold; not a clinical dose rule or residual-niche grade.
Kioi et al. (22)	Human glioblastoma xenografts in immunodeficient mice; 15 Gy × 1 or 2 Gy × 5; myeloid/recruitment blockade	Vascular recovery and recurrence perturbed (L3⁠–⁠L4). Human recurrent-tissue infiltration is observational.
Lu et al. (33)	Human presurgical antibody imaging at subtherapeutic doses; intact head and neck/pancreatic tissue	Spatial delivery association. Neither causal protection of residual cells nor therapeutic-dose efficacy established.
Powles et al. (7)	Randomized human adjuvant trial in ctDNA-positive muscle-invasive bladder cancer	Clinical benefit of molecular-risk-directed therapy. Does not identify or validate a spatial niche.

Treatment details refer to the cited experiments and are not clinical dose recommendations. L1–L4 refer to the biological evidence questions defined in the text, not grades of clinical efficacy. TAC, docetaxel, doxorubicin, and cyclophosphamide.

Single-cell RNA sequencing resolves states but loses tissue geography. Spatial transcriptomics, multiplexed imaging, and imaging mass cytometry define neighborhoods, boundaries, and local interactions. Lineage tracing and barcoding test ancestry; perturbation establishes causality. Single-cell spatial pharmacobiology demonstrates how drug distribution, target engagement, ECM, CAFs, vasculature, and immune cells can be co-measured in intact human tissue (33).

Analytical definitions should be prespecified. A niche should include a spatial rule, cell-state composition, treatment timepoint, and outcome or functional readout. Ligand-receptor inference alone is hypothesis-generating because transcript abundance does not prove ligand availability, receptor activation, or biological necessity. Results should be validated with protein localization, pathway activity, perturbation, or orthogonal assays.

### 7.2 Patient-derived models and causal testing

Epithelial organoids support malignant-cell perturbation; immune or stromal questions require deliberate co-culture or air–liquid-interface designs (45, 46). Tumor slices briefly retain native architecture (47). Patient-derived xenografts generally lack intact human immunity and can undergo host-specific evolution, whereas tumor-on-chip systems reproduce selected flow and gradient conditions in an engineered geometry (48, 49). Syngeneic mouse models preserve murine immunity but not human immune history, ligand–receptor compatibility, organ context, or prolonged latency. Residual sampling over days or weeks can resolve acute remodeling without reproducing multi-year human dormancy.

A CAF-mediated hypothesis needs fibroblast state and matrix; an immune-clearance hypothesis needs autologous antigen presentation and effector function; a drug-delivery hypothesis needs realistic geometry, perfusion, and exposure. Concordance between a patient specimen, a model that preserves the implicated interaction, and a selective perturbation is stronger than replication across several models that all omit the same compartment.

### 7.3 Circulating biomarkers and clinical surveillance

Serial ctDNA estimates molecular burden and can inform recurrence-risk assessment or timing of tissue investigation. A rise is not by itself proof of niche reactivation, and ctDNA-guided treatment does not establish niche-directed efficacy. Sensitivity varies with tumor type, burden, and anatomical compartment (50); a negative result cannot exclude viable cells with low shedding. Candidate niche biomarkers require linked tissue evidence and functional validation beyond prognostic association.

Integration with immune clonotypes, soluble proteins, metabolic measures, imaging, and spatial tissue signatures is a research strategy. A proposed niche-specific biomarker should be linked to the relevant interaction, respond to its perturbation, and change early enough to inform a defined decision. Increased assay complexity alone does not establish specificity.

## 8 Therapeutic interception of residual niches

### 8.1 Target a dependency, not a compartment label

A compartment label is too broad to define a treatment. A candidate target should instead be a state, interaction, or function required by residual cells, with a safety plan that accounts for the same compartment’s roles in normal repair, immune activation, or tumor restraint.

Mechanistic redundancy is a second challenge. When several growth factors, adhesion pathways, and matrix signals converge on the same residual state, blocking one mediator may leave the neighborhood functional. Alternatives include targeting a shared downstream dependency, improving regional drug delivery, exploiting a collateral metabolic vulnerability, or directing immunity against a state-specific antigen.

### 8.2 A proposed framework for treatment timing

We propose four windows as a way to organize research questions (**Figure 2**), not as a clinically validated classification applicable across tumors. Each requires a distinct endpoint and evidence that the targeted interaction is relevant at that interval.

**Figure 2:**
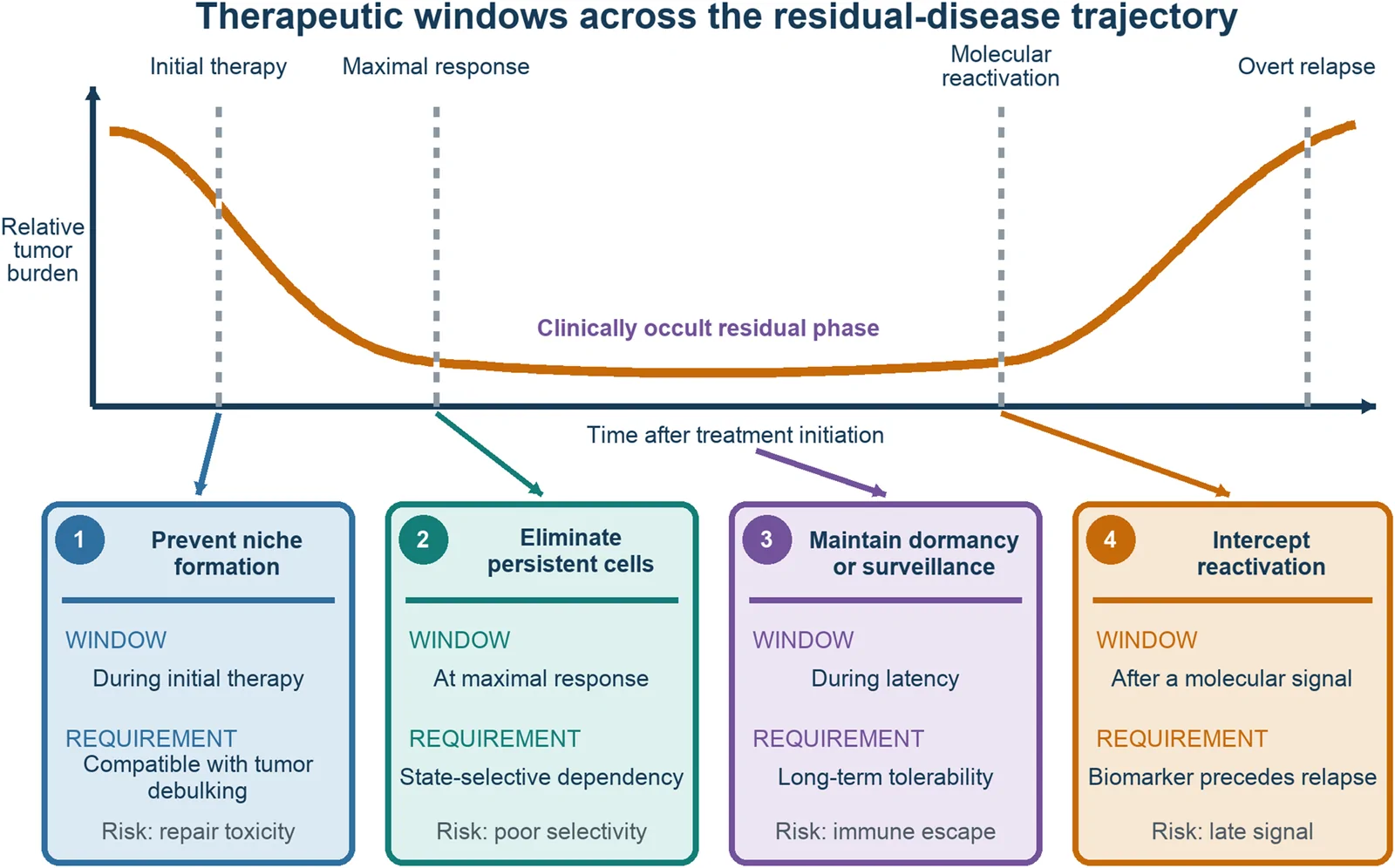
Proposed research windows across residual disease. The schematic curve illustrates one possible trajectory and does not represent measured kinetics or an obligatory sequence. Prevention during initial therapy, elimination near maximal response, maintenance of dormancy or surveillance, and interception after a molecular signal are proposed strategies rather than a validated clinical classification. Evidence and limitations differ by window, as discussed in the text. ctDNA can inform molecular-risk assessment but does not localize a tissue niche; a molecular signal may be absent or detected too late for interception.

During initial therapy, prevention of protective remodeling is supported by experimental stromal and myeloid perturbations (15, 22). The key limitation is that normal repair and beneficial immunity occur concurrently; a combination benefit does not isolate which temporal interaction was necessary.

For elimination near maximal response, GPX4 vulnerability and collateral targeting provide a mechanistic rationale, not validation of a clinical maximal-response schedule (3, 38). Selectivity over normal and immune cells, adequate regional exposure, and an effect on relapse require testing; low burden alone is not a therapeutic rationale.

During latency, vascular and adaptive-immune experiments show that dormant or occult populations can be restrained (36, 40). A maintenance intervention must preserve that restraint and remain tolerable over the relevant human interval. Experimental equilibrium does not establish a safe treatment for years of clinical dormancy.

After a molecular signal, ctDNA-guided adjuvant trials provide setting-specific clinical evidence for molecular-risk-directed treatment (6–8). They do not validate disruption of a spatial niche or prove that a signal always precedes radiographic relapse sufficiently early. This window requires an appropriate assay, sampling interval, and prospectively tested trigger.

### 8.3 Evidence standards for translation

Biological evidence and clinical validation should remain separate. The four niche evidence levels introduced above establish progressively stronger support for a local interaction; clinical utility additionally requires feasible target engagement and biomarker measurements, an acceptable safety profile, and controlled evidence of durable benefit. Even a level-4 mouse result remains preclinical until evaluated in the intended patient population.

Claims of biological failure require evidence that the intervention reached and modulated the relevant region. Poor delivery may leave the mechanism untested, but it still makes the intervention clinically ineffective. Trials should retain all enrolled patients in feasibility denominators, report failed biopsies and nonevaluable samples, and avoid analyses restricted to specimens with high tumor content.

Endpoints depend on setting. Pathological response and ctDNA clearance may be useful early measures, but relapse-free and overall survival remain necessary to prove durable benefit. In metastatic disease, time to regrowth after treatment withdrawal may be particularly informative for residual-state interventions. Biomarker-treatment interaction is stronger evidence than prognostic association alone.

## 9 Discussion and research priorities

The framework adds a treatment- and space-aware evidence map to descriptions of malignant-cell persistence. A cell-state signature, a nearby fibroblast, and a positive ctDNA assay answer different questions. Connecting exposure, localization, function, and relapse outcome helps identify which inference is justified and which experiment or clinical observation is still missing.

Temporal causality remains a priority. Many available studies lack paired pretreatment and post-treatment human tissue and cannot distinguish rare selected states from induced transitions. Longitudinal sampling should connect dose and timing to cell ancestry, regional drug exposure, spatial organization, and subsequent behavior. Observed disappearance of a state at relapse does not establish its earlier necessity.

Anatomical generalizability requires matching the model to the implicated organ and interaction. Human late recurrence can extend over decades, as shown in a population-based breast cancer cohort with follow-up to 32 years after diagnosis (51). Acute murine repair and short experimental dormancy do not reproduce that timescale, immune aging, or successive human treatments. Shared spatial signatures across species therefore support a hypothesis, not a common therapeutic effect.

A clinically trackable dependency is a further priority. Studies should distinguish prognostic, predictive, and pharmacodynamic biomarkers, specify the change relative to clinical relapse, and identify the decision that follows. The proposed windows become actionable only if the interaction is necessary at the relevant time and the intervention can safely reach and modulate it.

### 9.1 Conflicting and context dependent evidence

Pancreatic myofibroblast or Hedgehog-directed stromal depletion worsened outcomes in mouse models (31, 32), whereas selected fibroblast interactions protected lung cancer cells (3). Compartment abundance is consequently an inadequate treatment target. A stroma-rich region may instead mark underexposure or repair, as illustrated by low-dose human antibody mapping (33). Those alternatives require drug-distribution and functional measurements. Likewise, relapse histology can reflect regrown populations rather than the earlier residual reservoir (4); recurrence tissue alone cannot resolve which interaction sustained persistence.

### 9.2 Limitations

The purposive narrative search is vulnerable to selection and publication bias and cannot provide an exhaustive intervention comparison or effect estimate. Tumor types, metastatic sites, drug regimens, fractionation schedules, and sampling intervals differ substantially. Paired longitudinal human samples remain limited, and several mechanisms rely on cultures, engineered systems, or animal perturbations that omit human immunity or compress latency. Spatial sampling can miss rare reservoirs, and pharmacological effects may not isolate one compartment. Finally, molecular burden and prognostic association do not establish niche specificity; none of the proposed evidence levels or treatment windows has been validated as a universal clinical classification.

## 10 Conclusions

Residual disease can reflect interactions between surviving malignant cells and treatment-altered tissue, but the strength and necessity of those interactions vary by tumor, site, and regimen. Selected models reveal vulnerabilities enriched during persistence; this is a testable opportunity rather than a defining property of all residual disease.

Translation requires identifying the interaction, measuring regional exposure and modulation, and testing whether intervention improves durable outcome while preserving repair, surveillance, and tumor restraint. Spatial correlation, molecular detection, and clinical benefit must remain distinct until evidence connects them. The proposed framework supports that evaluation without assuming that the residual phase is always uniquely targetable.
